# Use of Remifentanil and Alfentanil in Endotracheal Intubation: A Comparative Study

**DOI:** 10.5812/kowsar.22287523.2130

**Published:** 2011-09-26

**Authors:** Farnad Imani, Mahmoud-Reza Alebouyeh, Zahra Taghipour Anvari, Seyyed Hamid Reza Faiz

**Affiliations:** 1 Department of Anesthesiology and Pain Medicine, Rasoul-Akram Medical Center, Tehran University of Medical Sciences (TUMS), Tehran, Iran

**Keywords:** Alfentanil, Remifentanil, Propofol, Endotracheal intubation

## Abstract

**Background:**

Opioids, such as alfentanil, are used to facilitate endotracheal intubation without the use of neuromuscular blocking agents in patients undergoing elective surgery.

**Objectives:**

The goal of this study was to evaluate the endotracheal intubation conditions when remifentanil or alfentanil was used with propofol without the application of neuromuscular blocking agents.

**Patients and Methods:**

One hundred American Society of Anesthesiologists (ASA) grade I patients scheduled for elective surgery were enrolled in this prospective, randomized, triple-blinded study. The patients were randomized to group A (alfentanil) or R (remifentanil). In group A, alfentanil (50 mcg/kg) was intravenously injected over 10 seconds, and after 45 seconds or at the occurrence of apnea, propofol (2 mg/kg) was intravenously injected over 5 seconds. Thirty seconds after the administration of propofol, laryngoscopy and endotracheal intubation were attempted. In group R, remifentanil (5 mcg/kg) was administered instead of alfentanil. Intubation conditions, including ease of laryngoscopy, patency of the vocal cords, jaw relaxation, limb movement (1-4 score), and also, demographic data were evaluated.

**Results:**

There were no demographic data differences between groups (age, weight, and sex). Further, laryngoscopy, jaw relaxation, and limb movement scores were similar in the R and A groups and there were no significant differences, but vocal cords were significantly more patent in group R than those in group A (P = 0. 028).

**Conclusions:**

The results of this study showed that remifentanil, similar to alfentanil, provided excellent conditions for endotracheal intubation when used with propofol for the induction of anesthesia; however, remifentanil improved the patency of the vocal cords to a greater extent than alfentanil.

## 1. Background

Endotracheal intubation is one of the measures conducted to maintain the airway while inducing anesthesia. Non-depolarizing muscle relaxants are generally administered to facilitate this procedure. However, in some situations, e. g. , a full stomach, difficult intubation, and certain neuromuscular diseases, the administration of these agents is considered controversial. Therefore, other agents and methods, such as opioid agents, intravenous (propofol instead of thiopental) or inhaled (sevofluran) hypnotics can be usedin such situations to facilitate endotracheal intubation (1-4). Opioids are agents that suppress respiration in addition to providing appropriate conditions for endotracheal intubation; they are therefore good substitutes for muscle relaxants when used along with intravenous or inhaled hypnotics during intubation (1, 2). Previous studies have examined the use of alfentanil for endotracheal intubation in children and adults without the use of muscle relaxants (5-10). Remifentanil is an ultra-short-acting opioid resulting in short-term complications (11-13).

## 2. Objectives

The aim of this study was to compare endotracheal intubation conditions using remifentanil (5 mcg/kg) with those using alfentanil (50 mcg/kg) while administrating propofol (2mg/kg) to induce anesthesia without using muscle relaxants in adult patients with normal airways (Mallampati grade I).

## 3. Patients and Methods

After approval of the Medical Ethics Committee and obtaining written informed consent, 100 adult ASA I patients (age, 20–50 years) scheduled to undergo elective surgery were enrolled in this prospective, triple-blinded study. The patients were randomly (using a computer-based randomizing table) assigned to group A (alfentanil) or group R (remifentanil) (50 in each group). Exclusion criteria were as follows:

1. Drug or alcohol abuse and smoking,
2. Airway assessed as Mallampati grade greater than I,
3. Full stomach

To achieve triple-blinding, the identity of the administered opioid was withheld from the physicians involved in drug administration, endotracheal intubation, and patient evaluation, and the person in charge of analyzing the data received them in an encrypted form.

After placing the patients on the operation bed and completing the physical examination, an intravenous (IV) line was set up, and 500 mL of ringer solution was infused. The patients were monitored for cardiac activity and heart rate by lead II electrocardiography, non-invasive blood pressure, heart rate and pulse oximetry. The dose of the opioid agent (500 mcg/mL alfentanil or 50 mcg/mL remifentanil in a 10 mL syringe) was prepared by an anesthesiologist blinded to study. Intravenous midazolam (1 mg) was injected before the administration of the other drug and 100% oxygen was given for 3 minutes. Then, IV alfentanil (50 mcg/kg) was slowly administered within 10 seconds. After 45 seconds or at the occurrence of apnea, propofol (2 mg/kg) was injected within 5 seconds, and after 30 seconds, laryngoscopy and endotracheal intubation was performed by another anesthesiologist (unaware of the opioid agent administered). After successful intubation, the drug necessary for maintaining anesthesia (propofol 100 and alfentanil 0. 5 mcg/kg/min) was administered. In group R, iv remifentanil 5 mcg/kg was administered instead of alfentanil during induction, and the rest of the procedure was the same as in control group. The following conditions of endotracheal intubation were assessed (Table 1):

- ease of laryngoscopy,
- patency of vocal cords,
- jaw relaxation, and
- limb movement, based on 1 to 4 scoring.

**Table 1.** Evaluation of the Quality of Endotracheal Intubation

	Score			
	1	2	3	4
Possibility of laryngoscopy	Simple	Fairly simple	Difficult	Impossible
Patency of vocal cords	Patent	With movement	Closing	Blocked
Jaw relaxation	Fully relaxed	Fairly relaxed	Moderate relaxation	Locked
Limb movement	Not moving	Little moving	Moderately moving	Entirely moving

### 3. 1. Statistical Analysis

Normal distribution of quantitative variables (age and weight) was evaluated by the Kolmogorov–Smirnov test. If the variables were normally distributed, the data were analyzed using a t-test. The collected data were expressed as the mean ± standard deviation. The qualitative (nominal) and ordinal variables (sex and intubation score) were assessed by the chi-square test, Mann-Whitney test, and Kendall’s Tau-b non-parametric correlations. Analytical studies were conducted using SPSS ver. 12 software. P > 0.05 were considered statistically significant.

## 4. Results

The distribution of age and weight variables was normal. Demographic data (age, weight, and sex) were not significantly different between the two groups (Table 2). As shown in Table 2, group A and group R did not differ significantly in mean age (32. 5 ± 8. 7 vs. 35 ± 9. 5 years; P = 0. 165) and mean weight (66 ± 9. 7 vs. 67. 6 ± 7. 3 kg; P = 0. 352). Male patients comprised 46% of group A and 58% of group R, indicating that the two groups did not differ significantly in this regard (P = 0. 230). As shown in Table 3, the scores for ease of laryngoscopy (A), jaw relaxation (C), and limb movement (D) did not differ significantly between the two groups (Mann-Whitney test), but vocal cord patency (B) in group A was significantly higher than that in remifentanil group (P = 0. 028). This means that the probability of movement of the vocal cords and their closing in group A was higher than that in group R.

**Table 2.** Demographic Data in the Studied Groups

	Alfentanil	Remifentanil	*P* value
Age ,y ^a^	32. 5 ± 8. 7	35 ± 9. 5	0. 165
Sex (M/F) ^a^	23/27	29/21	0. 23
Weight, Kg, Mean ± SD	66 ± 9. 7	67. 6 ± 7. 3	0. 352

^a^ No significant difference

**Table 3.** Comparison of Endotracheal Intubation Scores

	Alfentanil (%)	Remifentanil (%)	*P* value
Laryngoscopy score ^a^			0. 277
1	74	82	
2	20	18	
3	6	0	
4	0	0	
Vocal cords’ patency score ^b^			0. 028
1	64	84	
2	26	10	
3	8	6	
4	2	0	
Jaw relaxation score ^a^			0. 911
1	76	76	
2	18	22	
3	6	2	
4	0	0	
Limb movement score ^a^			0. 865
1	68	70	
2	24	20	
3	4	10	
4	4	0	

^a^ No significant difference

^b^ With significant difference

## 5. Discussion

In our study, following administration of remifentanil and propofol there was quite a suitable condition for endotracheal intubation in most of the patients. Endotracheal intubation following administration of remifentanil led to the same conditions as those using alfentanil, with better patency of the vocal cords.

Previous studies have shown that endotracheal intubation is possible without the use of muscle relaxants (1-4). Propofol administration alone can enable intubation in 60% of cases (4), and the addition of alfentanil increases the possibility to 86% in adults (3). Klemola’s study showed that remifentanil (at 3 and 4 mcg/kg) in combination with propofol afforded excellent intubation conditions (in comparison with alfentanil 30 mcg/kg) with success rates of only 55% and 20%, respectively, but the most suitable condition was created in 93% (14).

Various studies have indicated different success rates of intubation under desirable conditions, and these differences can be attributed to differences in the study design, scoring system for the intubation condition, the criteria defined for scoring, the dosage of the drugs, the order of administration of drugs, agents administered before the main drugs, the speed at which the drugs are injected (slow injection using an injection pump or fast injection), the duration of induction (90 seconds to 4 minutes), and the age of the patients (from infancy to adulthood). In a study by Alexander et al. , remifentanil (2 mcg/kg), alfentanil (50 mcg/kg), and succinylcholine (1 mg/kg) were administered after propofol, and excellent conditions for intubation were obtained in 35%, 85%, and 100% of the cases, respectively (15). The authors concluded that remifentanil is not as suitable for intubation as alfentanil and succinylcholine. However, in another study by the same group, remifentanil (3, 4, or 5 mcg/kg) was administered in combination with propofol and midazolam (injected before the other drugs), and excellent conditions were obtained in 60%, 95%, and 95% of the cases, respectively (16).

Tracheal intubation has also been performed with the use of intravenous hypnotics (propofol, thiopental, and etomidate) in some studies. These studies have revealed that propofol, in combination with remifentanil, is a suitable intravenous hypnotic for the induction of anesthesia and provides better intubation conditions compared to other intravenous hypnotics (1, 17). A study on the pharmacokinetic properties of remifentanil showed that propofol reduced the need for remifentanil required to suppress the patient’s reaction to laryngoscopy, thereby indicating a synergistic action between the two drugs (18). Similarly, remifentanil reduces the need for propofol during the induction of anesthesia (19). Collectively, these findings indicate that a combination of these two agents may be useful in inducing anesthesia for intubation. Remifentanil is 20- to 25-fold more potent than alfentanil, but has a shorter duration of action. This limited period of action is another advantage of remifentanil over alfentanil, which has the disadvantage of causing prolonged respiratory arrest when used in short surgeries (8).

The combination of remifentanil with propofol may also be advantageous in cases of long and difficult intubation, wherein it may not only be possible to inspect the airway with the laryngoscope but also assess whether the procedure can be continued or aborted in order to preclude the complications of prolonged respiratory arrest during anesthesia. This short period of respiratory arrest afforded with remifentanil is also beneficial in the case of infants, with the duration of respiratory arrest with the use of remifentanil (2 mcg/kg) and propofol (4 mg/kg) being similar to that with the use of succinylcholine (20). This combination is also useful in conditions when endotracheal intubation is necessary but muscle relaxation for surgery is not required, such as cases in which non-depolarizing muscle relaxants are contraindicated (e. g. , myopathy) and those in which succinylcholine is contraindicated despite the need for a fast endotracheal intubation (e. g. , hyperkalemia, burns, choline-esterase enzyme deficiency, or susceptibility to malignant hyperthermia). In addition, complications of depolarizing and non-depolarizing muscle relaxants and their antagonists and the resultant prolongation of the recovery period can be averted by avoiding their administration. A combination of remifentanil and propofol has been used for endotracheal intubation in patients of at different ages (children and adults) (1, 21-23), and the doses of 3 mcg/kg and 3 mg/kg for remifentanil and propofol, respectively, were found to provide desirable conditions for intubation (21). In adult patients who received iv midazolam and lidocaine before remifentanil (2 mcg/kg) and propofol (2 mg/kg) had better intubation conditions compared to those receiving thiopental (5 mg/kg) after 2. 5 minutes of induction: 85% of the propofol group and 50% of thiopental group had excellent conditions for intubation (23).

Our study was conducted on healthy young patients, and the outcome may be different in other population groups. The problems with this method and its limitations, as cited in a few studies, include its unsuitability for the elderly, decrease in intravascular volume, and its limitations in patients with cardiovascular or cerebrovascular diseases (24); since decrease in blood pressure induced in these patients might not be well tolerated. Further, intubation in such patients without the use of muscle relaxants might sometimes be dangerous; for example, if laryngoscopy and endotracheal intubation are conducted under inadequate conditions, they may cause injury to the airway or compromise ventilation. Thus, this method is best avoided in patients with high Mallampati grades or airway difficulties.

Remifentanil administration (1 to 3 mcg/kg) without propofol can lead to muscle stiffness (i. e. , difficult ventilation with a mask) depending on the drug dosage and the speed of administration (12, 13), but if administered in combination with propofol, muscle stiffness will rarely occur (20). Similarly, alfentanil (40 mcg/kg) in combination with propofol does not lead to significant muscle stiffness (8, 9).

Though high doses of short-acting opioids (such as remifentanil and alfentanil) may be avoided in outpatient procedures in order to prevent anesthetic-induced complications and enable earlier discharge, they can be used efficiently in hospitalized patients requiring prolonged surgery and in whom the use of muscle relaxants is considered controversial. On the other hand, due to its pharmacokinetic properties and the possibility of faster recovery (compared to alfentanil) and spontaneous return of respiratory functions (25), remifentanil appears to be the best choice opioid in such conditions. Notably, the use of this combination may be limited in elderly patients and subjects with low intravascular volume, because in such cases, high dosages of opioids may lead to bradycardia or a severe drop in blood pressure level. The combination may also be avoided in patients with Mallampati grades higher than I, i. e. , in those with airway difficulties, and those requiring short surgeries in whom the use of high opioid doses (especially alfentanil) delays discharge. In summary, we conclude that remifentanil in combination with propofol can be used for laryngoscopy and endotracheal intubation in young healthy patients to avoid the use of muscle relaxants.
